# *SETD2* variation correlates with tumor mutational burden and MSI along with improved response to immunotherapy

**DOI:** 10.1186/s12885-023-10920-4

**Published:** 2023-07-21

**Authors:** Xiaobin Zheng, Jing Lin, Jiani Xiong, Yanfang Guan, Bin Lan, Yi Li, Xuan Gao, Zhaodong Fei, Lisha Chen, Lizhu Chen, Ling Chen, Gang Chen, Zengqing Guo, Xin Yi, Weiguo Cao, Xinghao Ai, Chengzhi Zhou, Xiaofeng Li, Jun Zhao, Xiangtao Yan, Qitao Yu, Lu Si, Yu Chen, Chuanben Chen

**Affiliations:** 1 Department of Radiation Oncology, Clinical Oncology School of Fujian Medical University, Fujian Cancer Hospital, Fuzhou, 350014 Fujian Province China; 2 Department of Medical Oncology, Clinical Oncology School of Fujian Medical University, Fujian Cancer Hospital, Fuzhou, 350014 Fujian Province China; 3 Cancer Bio-Immunotherapy Center, Clinical Oncology School of Fujian Medical University, Fujian Cancer Hospital, Fuzhou, 350014 Fujian Province China; 4 Geneplus-Beijing Institute, Beijing, China; 5 Department of Pathology, Clinical Oncology School of Fujian Medical University, Fujian Cancer Hospital, Fuzhou, 350014 Fujian Province China; 6 Department of Radiation Oncology, Ruijin Hospital, Shanghai Jiao Tong University School of Medicine, Shanghai, China; 7 Department of Shanghai Lung Cancer Center, Shanghai Jiao Tong University Affiliated Chest Hospital, Shanghai, China; 8 First Affiliated Hospital, Guangzhou Medical University, Guangzhou, Guangdong Province China; 9 Department of Oncology, Affiliated Quanzhou First Hospital of Fujian Medical University, Quanzhou, Fujian Province China; 10 Department of Thoracic Oncology, Peking University Cancer Hospital & Institute, Beijing, China; 11 Department of Internal Medicine, The Affiliated Cancer Hospital of Zhengzhou University, Henan Cancer Hospital, Henan Province, Zhengzhou, China; 12 Department of Oncology, The Cancer Hospital of Guangxi Zhuang Autonomous Region, Nanning, Guangxi Zhuang Autonomous Region China; 13 Department of Renal Cancer and Melanoma, Peking University Cancer Hospital and Institute, Beijing, China

**Keywords:** Immune checkpoint inhibitors, Tumor mutation burden, Microsatellite instability, Tumor microenvironment, *SETD2* mutation

## Abstract

**Background:**

*SETD2* protects against genomic instability via maintenance of homologous recombination repair (HRR) and mismatch repair (MMR) in neoplastic cells. However, it remains unclear whether *SETD2* dysfunction is a complementary or independent factor to microsatellite instability-high (MSI-H) and tumor mutational burden-high (TMB-H) for immunocheckpoint inhibitor (ICI) treatment, and little is known regarding whether this type of dysfunction acts differently in various types of cancer.

**Methods:**

This cohort study used multidimensional genomic data of 6726 sequencing samples from our cooperative and non-public GenePlus institute from April 1 through April 10, 2020. MSIsensor score, HRD score, RNAseq, mutational data, and corresponding clinical data were obtained from the TCGA and MSKCC cohort for seven solid tumor types.

**Results:**

A total of 1021 genes underwent target panel sequencing reveal that *SETD2* mutations were associated with a higher TMB. *SETD2* deleterious mutation dysfunction affected ICI treatment prognosis independently of TMB-H (*p* < 0.01) and had a lower death hazard than TMB-H in pancancer patients (0.511 vs 0.757). Significantly higher MSI and lower homologous recombination deficiency were observed in the *SETD2* deleterious mutation group. Improved survival rate was found in the MSKCC-IO cohort (*P* < 0.0001) and was further confirmed in our Chinese cohort.

**Conclusion:**

We found that *SETD2* dysfunction affects ICI treatment prognosis independently of TMB-H and has a lower death hazard than TMB-H in pancancer patients. Therefore, *SETD2* has the potential to serve as a candidate biomarker for ICI treatment. Additionally, *SETD2* should be considered when dMMR is detected by immunohistochemistry.

**Supplementary Information:**

The online version contains supplementary material available at 10.1186/s12885-023-10920-4.

## Background

In the past decade, the use of immune checkpoint inhibitors (ICIs) has revolutionized the treatment of a variety of tumors, including lung cancer, melanoma, colorectal cancer, and renal cell carcinoma [1, 2]. However, only some tumors, such as Hodgkin's lymphoma and tumors with high microsatellite instability (dMMR or MSI-H), respond well to ICIs with an objective response rate (ORR) of 53–87%; the ORR of most tumors, such as non-small cell lung cancer, head and neck tumors, gastroesophageal tumors, bladder urothelial tumors, renal cell carcinoma, and hepatocellular carcinoma, is much lower (15–25%) [3, 4]. Meta analysis results showed that the incidence of grade 3–4 immunotoxicity was approximately 10–24% with programmed cell death protein 1( PD-1) or cytotoxic T lymphocyte-associated antigen-4 (CTLA-4) monotherapy [5, 6], and reached 59% with combined immunotherapy [7]. Therefore, identifying prognostic biomarkers is urgently needed to optimize patient benefits, minimize toxicity risk, and guide clinical approaches. Among all prognostic biomarkers, the first FDA approved biomarker was PD-L1; however, PD-L1 shows low negative predictive value, dynamic changes in expression, and lack of a confirmed threshold between different detection method [8]. Another FDA approved and promising biomarker was MSI-H detected by polymerase chain reaction or dMMR detected by immunohistochemistry, and patients with this biomarker showed an ORR rate of 53% to immunotherapy [3]. Regretfully, MSI-H mainly occurred in endometrial cancer, colorectal cancer, and gastric cancer with rates of just 31.37%, 19.72%, and 19.09%, respectively [9]. Subsequently, the FDA approved pembrolizumab for adults and children with TMB-H solid tumors; however this is applicable to only 13% of solid tumors and the ORR was limited to about 29% [10]. Given these limitations, the aforementioned biomarkers do not guarantee therapeutic benefits. An increasing number of studies have shown that patients with somatic alterations to genes involved in DNA damage repair (DDR), such as BRCA1 DNA repair associated (*BRCA1*), BRCA2 DNA repair associated (*BRCA2*); mismatch repair (MMR) genes including *MSH2* (mutS homolog 2), *MSH6* (mutS homolog 6), *MLH1* (mutL homolog 1), and *PMS2* (PMS1 homolog 2); DNA polymerase epsilon, catalytic subunit (*POLE*) and the DNA polymerase delta 1, catalytic subunit (*POLD1*) are more likely to elicit a durable anti-tumor immune response from ICI treatment, demonstrating an important role or DDR-related genes as prognostic biomarkers [4, 11–14].

The SET domain containing 2 histone lysine methyltransferase (*SETD2)* gene encodes the functional domain of an enzyme that trimethylates histone H3 at lysine 36 (H3K36me3), which mediates MMR in a way that removes lesions associated with a persistently open chromatin structure in early replication, and preferentially safeguards active transcripts during replication by recruiting hMutSa which can quickly identify the mismatch to initiate the MMR reaction [15, 16]. *SETD2* also favors homologous recombination repair (HRR) via activation of ataxia-telangiesctasia mutated (*ATM*) and formation of *RAD51* presynaptic filaments upon DNA double-strand breaks [17]. Loss of *SETD2* function may afford an alternative mechanism for p53-mediated checkpoint inactivation and a strong reduction in DDR, resulting in alterations in DNA regulation, increased spontaneous mutations, and chromosomal instability [18]. Deleterious mutations in *SETD2* have been implicated in a wide range of solid tumors, including renal cancer, lung cancer, melanoma, gastrointestinal cancer, and endometrial cancer [19, 20]. Given the preclinical evidence, whether *SETD2* dysfunction was a complementary or independent factor to MSH-H and TMB to predict the prognostic of ICIs treatment needed to be figure out and whether this kind of dysfunction acted differently in different cancer types warrants further study. So this study was conducted. We inferred that patients with *SETD2* deficiency may benefit from immunotherapy through higher-TMB, more unstable microsatellites or other mechanism and we test this hypothesis in Chinese population from our corroborative GenePlus institute and the American population from TCGA [21] and MSKCC [22, 23] cohort.

## Methods

### Patients and samples

We used a GenePlus-Beijing Clinical Sequencing cohort from March 2018 to April 2020 to evaluate 6726 Chinese pan-cancer patients (Table S1). Among them, 375 patients with *SETD2* mutations were identified. Upon reviewing their treatment, response, and overall survival, 362 patients were excluded due to 1) only receiving one to three cycles of ICIs treatment, 2) incomplete imaging data, 3) incomplete follow-up data, or 4) a benign tumor. The remaining 13 patients receiving ≥ 4 cycles of ICIs treatment from nine cancer centers were selected for survival analysis. A customized panel of 1021 genes (Table S2) were sequenced in the GenePlus cohort containing whole exons. In addition, selected introns of 288 common driver genes and high frequency mutant regions recorded in the Catalogue of Somatic Mutations in Cancer (COSMIC, http://cancer.sanger.ac.uk/cosmic) were added for 733 genes.

### Prediction of the functional impact of mutations

We used the functional impact predicting tools Sorting Intolerant From Tolerant (SIFT) and Polymorphism Phenotyping v2 (PolyPhen-2), which were integrated in the Ensembl Variant Effect Predictor (http://uswest.ensembl.org/info/docs/tools/vep/index.html), to predict the effects of missense mutations on protein function. A mutation with a SIFT score < 0.05 was predicted to be deleterious; PolyPhen-2 scores of > 0.9 and 0.447–0.9 were considered as probably damaging and possibly damaging, respectively. In this study, a deleterious mutation was defined as any form of the following: deleterious mutations predicted by SIFT, a possibly/probably damaging mutation predicted by PolyPhen-2, a nonsense mutation, a stop-gain mutation, a frameshift deletion, a frameshift insertion, an inframe deletion, or an inframe insertion. Any gene with a deleterious mutation was marked as mut + , and those without a genomic alteration or deleterious mutation were marked as mut-.

### Tumor mutation burden analysis

TMB was defined as the total nonsynonymous mutation counts after filtering by t_alt/(t_alt + t_ref) ≥ 0.05. In the GenePlus cohort, nonsynonymous mutations included missense, cds-del, cds-ins, cds-indel, frameshift, nonsense, stop-gain, stop-retain, and init-loss. In the MSKCC-IMPACT [24] and The Cancer Genome Atlas [21] (TCGA) cohort, nonsynonymous mutations included frameshift_del, frameshift_ins, inframe_del, inframe_ins, missense mutation, nosense_mutation, nonstop mutation, and translation_start_site mutations. A frameshift in the Memorial Sloan Kettering Cancer Center (MSKCC) database was also included. In the POPLAR/PAK cohort, nonsynonymous mutations included missense and nonsense mutations. In the present study, the upper quartile of TMB was deemed as TMB-high (TMB-H).

Comparisons were done between *SETD2* mut + , BRCA1/2 mut + (*BRCA1* or BRCA2 deleterious mutations), MMR genes mut + (*MLH1, MSH2, MSH6*, or *PMS2* deleterious mutations), POLE/D1 mut + (*POLE* or *POLD1* deleterious mutations), and a None group (without *SETD2*, *BRCA1, BRCA2, MLH1, MSH2, MSH6, PMS2, POLE*, or *POLD1* deleterious mutations) using the Wilcoxon rank-sum test by R (version 3.6.0).

### MSIsensor and HRD score

MSIsensor [25] is a tool developed for determining the MSI status by quantifying the percentage of unstable microsatellites. We downloaded the MSIsensor scores of TCGA pan-cancer samples from Mandal R. et al. [26]. A MSIsensor score ≥ 4 was denoted as MSH-H [27, 28]. HRD is the functional defect in HRR. In our study, we downloaded the HRD score of TCGA patients from a published article [29]; the HRD score was equal to the unweighted numeric sum of the number of subchromosomal loss of heterozygosity (LOH) regions longer than 15 Mb, the number of regions of allelic imbalance that extended to one of the subtelomeres but did not cross the centromere [the number of telomeric allelic imbalances (TAIs)], and the number of break points between regions longer than 10 Mb after filtering out regions shorter than 3 Mb [large-scale state transitions (LSTs)]. The higher the HRD score, the more abnormal the homologous recombination repair.

### CIBERSORT analysis using TCGA data

The CIBERSORT [30] algorithm (https://cibersort.stanford.edu/) was used to quantify the relative fraction of 22 immune cell types by mRNA expression data. In this study, 3702 TCGA samples from seven cancer types were uploaded for estimation of the abundance of cell types. After estimation, 2360 output cases with *P*-values < 0.05 were included for further Wilcoxon rank-sum test analysis.

### ESTIMATE analysis using TCGA data

The ESTIMATE [31] algorithm was used to quantify the immune and stromal components in a tumor tissue by uploading mRNA expression data. In this study, the ESTIMATE package in R (version 3.6.0) and 3702 TCGA mRNA expression datasets from seven cancer types were used to calculate stromal, immune, and estimate scores. Immune scores were further analyzed by Wilcoxon rank-sum test to compare the differences in immune scores between *SETD2* mut + groups and *SETD2* mut- groups.

### Different gene expression profiles

There were 3892 transcripts per million (TPM) datasets from patients with seven cancer types including bladder urinary cancer, colorectal adenocarcinoma, renal carcinoma, non-small cell lung cancer (NSCLC), skin cutaneous melanoma, stomach adenocarcinoma, and endometrial carcinoma in the TCGA cohort that were downloaded from https://www.cbioportal.org/. The mRNA expression from cBioPortal was quantified by RNA seq by expectation–maximization (RSEM) [32] and was log-transformed as log2 (data + 1) for Wilcoxon rank-sum test analysis.

### Gene set enrichment analysis

Gene set enrichment analysis (GSEA) is a computational method that determines whether the expression of a predefined gene set differs significantly between two phenotypes. There are three key elements in the GSEA method including the enrichment score (ES), the nominal *P* value, and a normalized enrichment score (NES). The ES is the primary statistic for examining gene set enrichment results and the NES is used to normalize the ES account according to the size of the set. The nominal *P* value estimates the statistical significance of the ES, with a nominal *P* < 0.05 considered statistically significant. Single sample GSEA (ssGSEA) [33] is an extension of GSEA that works at the level of one sample each time rather than the whole population, as with the GSEA that determines whether the expression of a predefined gene set differs significantly between two phenotypes. The enriched score represented the degree to which our input gene set was up or downregulated in each corresponding sample.

### Statistical analysis

Survival curves were delineated by the Kaplan–Meier method, and the Log-rank method was used to assess the significance between groups. Univariate and multivariate Cox regression analyses were implemented to calculate the hazard ratio on progression-free survival (PFS) and overall survival (OS). Multivariate binary logistic regression was used to analyze the influencing factors of TMB-H. If TMB, TPM, immune scores, or 22 immune cell fractions estimated by CIBERSORT were normally distributed, a Student’s t-test was used to determine the differences between two groups; otherwise, the Wilcoxon rank-sum test was used. Wilcoxon rank-sum test analyses were conducted by R-3.6.0 [34], and others by GraphPad Prism (version 8.0.1) or SPSS version 25.0 (SPSS, Inc.). All tests were two-sided and all reported P values did not adjust for multiple comparisons, with *P* < 0.05 denoted as statistically significant.

## Results

### The prevalence of SETD2 mutations in pan-cancer

**Fig.1:**
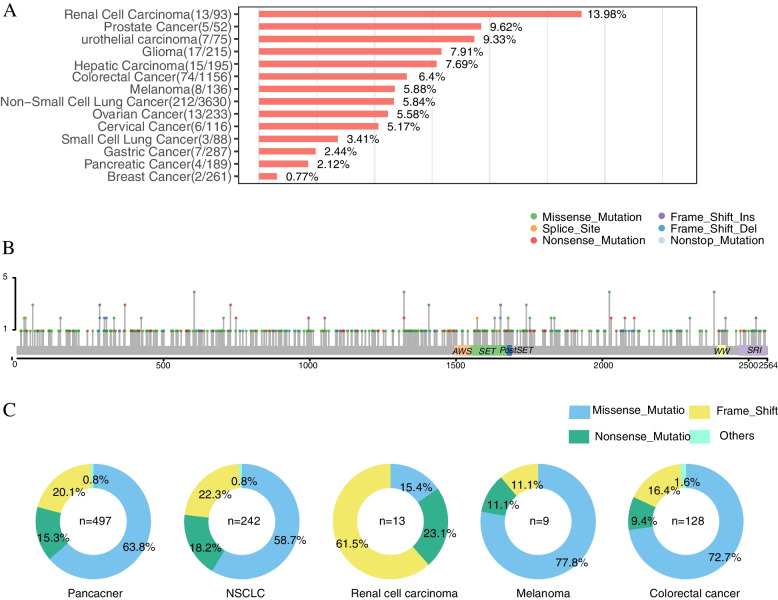
**A** Incidence of *SETD2* deleterious mutations in pan-cancer. Red, green, and blue bars represent the frequency of *SETD2* nonsynonymous mutations in pan-cancer in the Geneplus cohort. (Nonsynonymous mutations include frameshift indels, inframe indels, missense mutations, nonsense mutations, nonstop mutations, and translation start site mutations.) **B** The domains and the corresponding mutation sites of *SETD2* mutations. Domains and the corresponding mutation sites of the *SETD2* gene identified in the Geneplus cohort. Green, red, blue, brown, orange, and purple dots represent Missense_Mutation, Nonsense_Mutation, Frame_Shift_Del, In_Frame_Del, Splice_Site, and Frame_Shift_Ins, respectively. **C** Proportion of *SETD2* mutation forms by cohort and cancer type

To examine the occurrence of *SETD2* mutations in different cancers, we first evaluated the *SETD2* mutational prevalence (Fig. 1A). A total of 6726 samples were analyzed and the results showed that renal cell carcinoma had the highest *SETD2* mutational frequency (13/93; 13.98%) followed by prostate cancer (5/52; 9.62%), urothelial carcinoma (7/75; 9.33%), glioma (17/215; 7.91%), hepatic carcinoma (15/195; 7.69%), colorectal cancer (74/1156; 6.4%), melanoma (8/136; 5.88%), and NSCLC (203/3630; 5.84%). The Fig. 1B was drawn with R’ “maftool” package, which showed the mutation sites and mutation types in a given gene. Further statistical analysis revealed non-hotspot mutations within *SETD2* (Fig. 1B) with missense mutations predominating in NSCLC, melanoma, and colorectal cancer, whereas truncating mutations predominated in renal cell carcinoma (Fig. 1C). The functional impact predicting tools SIFT and PolyPhen-2 were used to predict deleterious missense mutations; samples with and without *SETD2* deleterious mutations were marked as *SETD2* mut + and *SETD2* mut-, respectively.

### The correlation between SETD2 deleterious mutation and tumor mutation burden

**Fig. 2:**
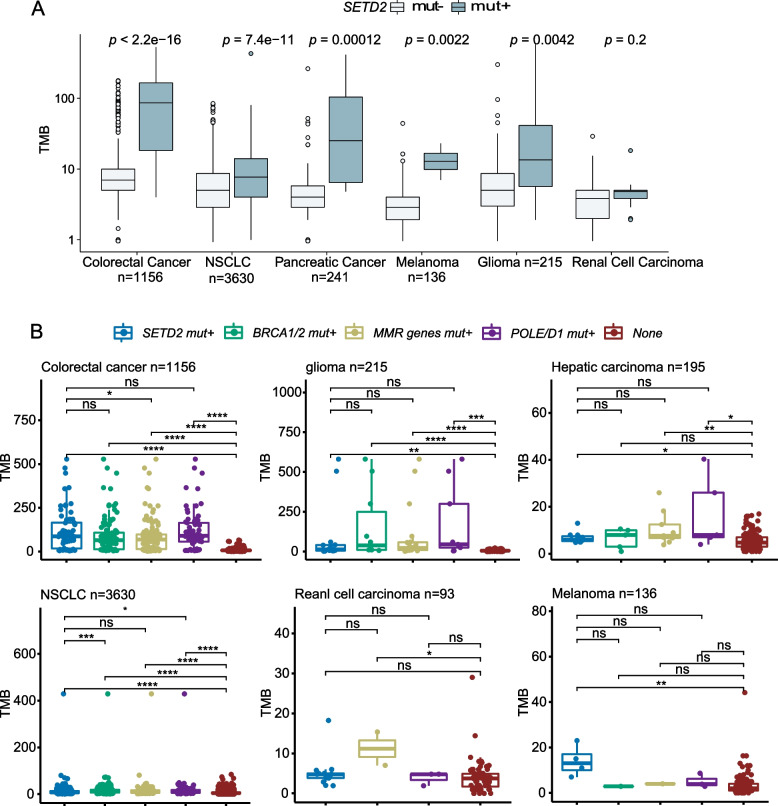
**A** Correlation between *SETD2* deleterious mutations and TMB. The difference of TMB in patients with *SETD2* deleterious mutations and non-deleterious mutations across different cancer types in the GenePlus cohort. **B** Difference of tumor mutation burden (TMB) in patients with diverse molecular features (*SETD2* deleterious mutations, *BRCA1/2* deleterious mutations, *MMR* genes deleterious mutations, or *POLE/D1* deleterious mutations) from the GenePlus cohort (Wilcoxon rank-sum test; ****, *P* < 0.0001; ***, *P* < 0.001; **, *P* < 0.01; *, *P* < 0.05.)

Since *SETD2* is a crucial element for maintaining genomic stability [35], we assumed that a *SETD2* deleterious mutation may lead to higher levels of TMB. The TMB of a total of 6721 patients form different cancer types was calculated and the TMB was compared between *SETD2* mut + and *SETD2* mut- groups. We found that the TMB levels in different tumors were stratified by *SETD2* mutation status. We observed a significantly higher TMB level in *SETD2* mut + in colorectal cancer (*P* < 0.001), NSCLC (*P* < 0.001), melanoma (*P* = 0.0022), glioma (*P* = 0.0042), and pancreatic cancer (*P* = 0.00012) (Fig. 2A). As TMB is considered a favorable biomarker for ICI treatment, it is highly likely that *SETD2*mutations can also serve as a prognostic factor for ICIs therapy [36].

To evaluate the effects of DDR-related mutations on TMB status, we further examined the TMB level in tumors with such mutations. The results showed no significant difference in TMB in samples comparing *SETD2* mutants to *BRCA1/2,* MMR, and *POLE/D1* mutant genes in colorectal cancer, glioma, hepatic carcinoma, NSCLC, renal cell carcinoma, and melanoma (Fig. 2B). Given that tumor mutation load is a consequence of the rates of mutagenesis and DDR, higher TMB and activation of the DDR system might be consistent with a more drastic mutagenesis in patients with deleterious *SETD2* mutations. We additionally investigated the change to DDR pathways resulting from *SETD2* dysfunctional mutations. The results showed upregulation in DDR pathways including HRR, MMR, nucleotide excision repair (NER), Fanconi anemia pathway (FA), translesion DNA synthesis (TLS), nonhomologous end-joining (NHEJ), and checkpoint factors (CPF), demonstrating a compensatory activation in DDR pathways due to a *SETD2* mutation (Figure S1).

### The correlation between SETD2 deleterious mutations and microsatellite status

**Fig. 3:**
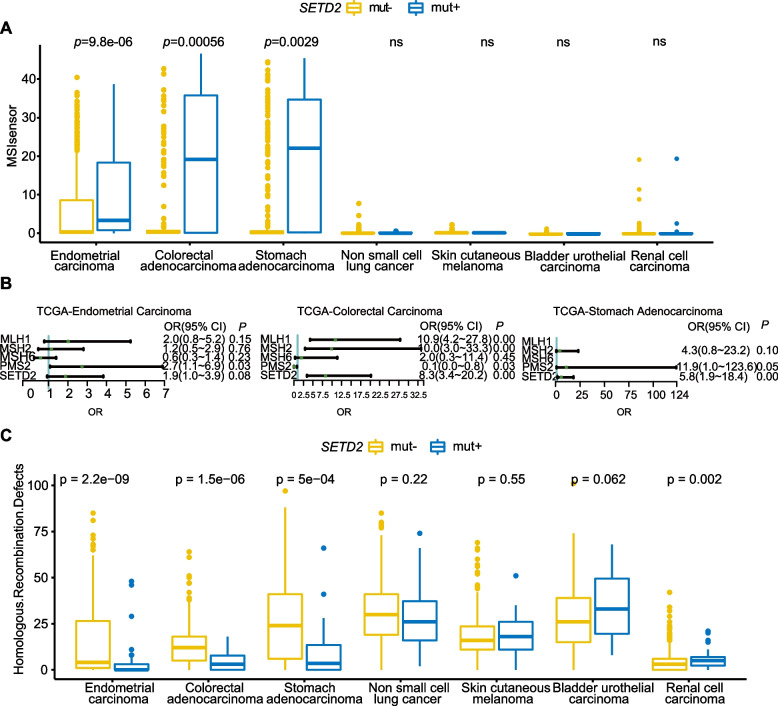
Correlation between *SETD2* deleterious mutations, MSI, and HRD. **A** Differences in MSIsensor scores in patients with *SETD2* deleterious mutations and non-deleterious mutations in the TCGA cohort across seven cancer types. **B** Odds ratios and 95% confidence intervals calculated by multivariate binary logistic regression comparing the risk of MSI-H (MSIsensor score ≥ 4) for patients with and without mutated genes in endometrial carcinoma, colorectal carcinoma, and stomach adenocarcinoma. **C** Differences in HRD scores in patients with *SETD2* deleterious mutations and non-deleterious mutations in the TCGA cohort across seven cancer types

As microsatellite status represent the phenotype of genomic instability, we next investigated the correlation between *SETD2* deleterious mutations and replication slippage variants at microsatellite regions. MSIsensor [25] is a tool developed for determining the MSI status by quantifying the percentage of unstable microsatellites. We downloaded the MSIsensor scores of TCGA pan-cancer samples from Mandal R. et al. [26]. A MSIsensor score ≥ 4 was denoted as MSH-H [27, 28]. Wilcoxon rank-sum tests showed that the *SETD2* mut + group had a significantly higher MSIsensor score than that the *SETD2* mut- group in endometrial carcinoma, colorectal adenocarcinoma, and stomach adenocarcinoma (Fig. 3A). Since *MSH2, MSH6*, *MLH1*, and *PMS2* are critical for MMR, mutations in any of these genes can cause MSI-H. We then evaluated MSIsensor scores among *MLH1* mut + , *MSH2* mut + , *MSH6* mut + , and *PSM2* mut + groups and compared to the *SETD2* mut + group (Figure S2A). The results showed no significant differences in MSIsensor score between patients who bore alterations within these genes. Further analysis showed that a *SETD2* deleterious mutation was an independent factor influencing MSI-H in colorectal carcinoma (*P* < 0.0001) and stomach adenocarcinoma (*P* = 0.003) (Fig. 3B). Preclinical data showed that *SETD2*-dependent histone H3K36 trimethylation is required for homologous recombination repair and genome stability [17]. However when exploring the potential effects of a *SETD2* mutation on homologous recombination pathways, we observed a decreased HRD score in the *SETD2* mut + group compared to the *SETD2* mut- group in endometrial carcinoma, colorectal adenocarcinoma, and stomach adenocarcinoma. (Fig. 3C).

### The correlation between SETD2 deleterious mutations and an inflamed tumor microenvironment

**Fig. 4:**
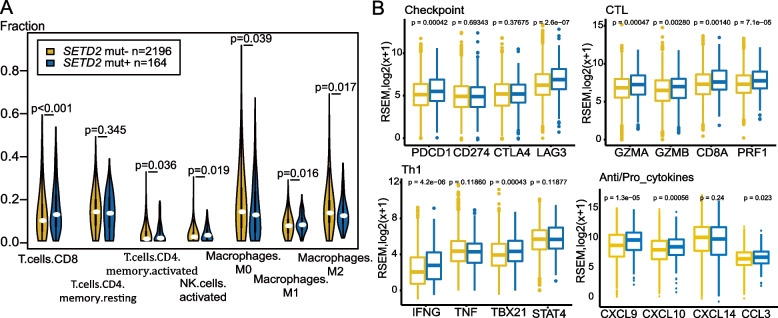
Tumors with *SETD2* deleterious mutations exhibit an inflamed immune microenvironment. **A** Different fractions of seven types of immune cells estimated by the CIBERSORT deconvolution algorithm between *SETD2* deleterious mutation and *SETD2* non-deleterious mutation groups. Samples were taken from patients across seven types of cancer in the TCGA cohort (bladder urinary cancer, colorectal adenocarcinoma, renal carcinoma, NSCLC, skin cutaneous melanoma, stomach adenocarcinoma, and endometrial carcinoma) (*N* = 2360). **B** Comparison of the expression of immune-related gene profiles between the *SETD2* non-deleterious mutation group and the deleterious mutation group. Samples were taken from patients across seven cancer types in the TCGA cohort (bladder urinary cancer, colorectal adenocarcinoma, renal carcinoma, NSCLC, skin cutaneous melanoma, stomach adenocarcinoma, and endometrial carcinoma)

A *SETD2* mutation tends to cause a higher level of TMB and MSI, which may lead to an inflamed immune signature. Therefore, we next estimated the fraction of infiltrating immune cells as well as immune and stromal components using the CIBERSORT (Fig. 4A) algorithm and ESTIMATE, respectively, to analyze the TCGA database. The results exhibited a significantly increased fraction of memory and activated CD8 and CD4 T-cells, activated NK cells and M1 macrophages, and a significant decrease in M0 and M2 macrophages in the *SETD2* mut + group (Fig. 4A). There was also a higher immune score in the *SETD2* mut + group in colorectal adenocarcinoma, renal cell carcinoma, and endometrial carcinoma (Figure S3). Immune gene clusters were upregulated, including checkpoint (*PDCD1* and *LAG3*), cytotoxic lymphocyte (*GZMA, GZMB, CD8A*, and *PRF1*), Th1 (*IFNG* and *TBX21*), anti-tumor chemokines (*CXCL9*), and pro-tumor chemokines (*CCL3*) (Fig. 4B, Table S3). This revealed that a *SETD2* deleterious mutation was associated with a more inflamed tumor microenvironment in patients with renal cell carcinoma (Figure S4A), colorectal adenocarcinoma (Figure S4B), and endometrial carcinoma (Figure S4C).

### The correlation between deleterious SETD2 mutations and response to ICIs

**Fig. 5:**
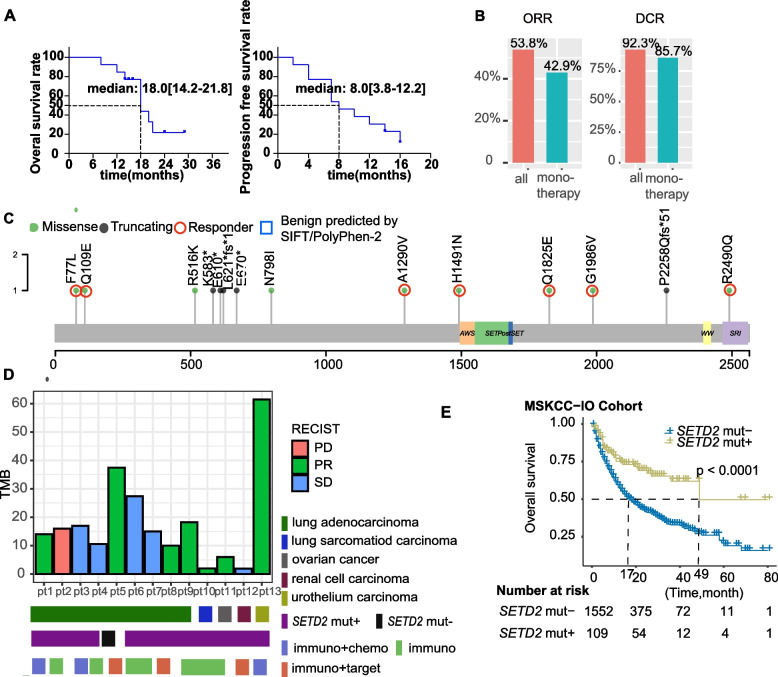
**A** Overall survival (OS) and progression free survival (PFS) curves of immune checkpoint inhibitor (ICI)-treated patients. **B** ORR rates and DCR rates of patients with PD-1/PD-L1 inhibitor monotherapy, or combined therapy with other drugs. **C** Mutation sites and types of *SETD2* mutation in ICI-treated patients. **D** Response evaluation criteria in solid tumors (PR, partial response; SD, stable disease; PD, progression disease), cancer types, *SETD2* mutation status, and treatment types of GenePlus ICI-treated patients. **E** *SETD2* deleterious mutations are linked with improved survival outcome in the ICI-treatment cohort

Herein, we investigate the survival outcome of patients with or without *SETD2* mutations who underwent ICI treatment. A 6726 Chinese pan-cancer population with 375 patients who had *SETD2* mutations was retrospectively analyzed (Table S1). Unfortunately, 362 patients were excluded due to 1) only receiving one to three cycles ICIs treatment, 2) incomplete imaging data, 3) incomplete follow-up data, or 4) a benign tumor. The remaining patients who met our criteria had been diagnosed with lung adenocarcinoma (9/13), lung sarcomatous carcinoma (1/13), ovarian cancer (1/13), renal cell carcinoma (1/13), or urothelium carcinoma (1/13) (Table S4). Among these patients, seven were treated with mono-ICIs, three were treated with ICIs and chemotherapy, and three were treated with ICIs and target therapy. The disease course of two patients showed an exceptional response to ICI treatment, as shown in Figure S5. The median OS and PFS of the 13 patients were 18 months and 8 months (Fig. 5A), respectively, or 18 months and 7 months, respectively, for PD-1/PD-L1 monotherapy. The overall response rates (ORRs) were 42.9% (3/7) for PD-1/PD-L1 monotherapy and 53.8% (7/13) for all patients (Fig. 5B), which is better than ORR for non-selective cancer patients (15%–35%) [37–42]. The disease control rates (DCRs) were 92.3% (12/13) for all patients and 85.7% (6/7) for PD-1/PD-L1 monotherapy (Fig. 5B). The mutational sites were scattered throughout the whole gene (Fig. 5C), and the details are shown in Fig. 5D. This phenomenon was validated by the results, which revealed a significantly better (*P* < 0.0001) OS in *SETD2* mut + than that in *SETD2* mut- patients (Fig. 5E). Multivariate analysis with the adjustment for TMB, *MMR* gene mutations, and *POLE/D1* mutations also showed that *SETD2* deleterious mutations were an independent factor influencing ICI efficacy (Table S5) and the hazard ration was much lower in *SETD2* deleterious mutation group than TMB-H (0.511 vs 0.757). These results indicate a potential tissue prognostic role of *SETD2* deleterious mutations in ICI-treated patients.

## Discussion

This study aimed to determine whether *SETD2* dysfunction is a complementary or independent factor of MSH-H and TMB, and whether this type of dysfunction acts differently in various types of cancer.

In this study, we investigated the mutation distribution in detail and found that mutations were scattered throughout the *SETD2* gene without accumulating in any specific area. To better understand the impact of *SETD2* dysfunction, analysis of cases with deleterious *SETD2* mutations were applied in this study.

As a DDR-related gene, *SETD2* ensures precise DNA replication. Accumulating evidence had shown that mutations in DDR-related genes, such as *MSH2, MSH6, PMS2, MLH1, BRCA1/2,* and *POLE/D1*, which function in MMR, HRR, and BER, are the main reasons for higher TMB and an immunogenic response [43–46]. In our study, we found a parallel tendency of a TMB increase between *SETD2* deleterious mutations and DDR gene mutations including *BRCA1/2, MMR* genes, and *POLE/D1* across seven cancer types*.* Our multi-cox regression analysis showed that *SETD2* deleterious mutations (distinct from *BRCA1/2, MMR* genes, and *POLE/D1*) were an independent factor influencing OS with adjustment for TMB, and an even lower death hazard ratio following ICI treatment was found in the *SETD2* deleterious mutation group compared with TMB-H patients. The deletion of *SETD2* can result in accumulation of DNA damage and impaired cellular tolerance towards replication stress through either loss of *SETD2* function or dysregulation of recruitment and activation of early DDR factors like *ATM*, *p53*, and *RAD51*, explaining the correlation between *SETD2* mutation and TMB elevation [18].

FDA approved MSI-H detected by polymerase chain reaction or dMMR detected by immunohistochemistry with any deficiency of MSH2, MSH6, PMS2 and MLH1 as biomarker for immunocheckpoint treatments. Preclinical data showed that in Hela cells, H3K36me3 converted by *SETD2* from H3K36me2 recruits hMutSa onto chromatin via its interaction with the hMSH6 PWWP domain before DNA replication initiates. During DNA replication, H3K36me3-PWWP interaction was disrupted and released hMutSa which can quickly identify the mismatch to initiate the MMR reaction [16]. We found that *MSH2*, *MSH6*, *MLH1*, *PMS2* and *SETD2* independently influencing MSI-H in endometrial carcinoma, colorectal adenocarcinoma, and stomach adenocarcinoma patients. This finding was a confirmation of preclinical data and identify a distinct role of *SETD2* between endometrial carcinoma, colorectal adenocarcinoma, stomach adenocarcinoma and bladder urinary cancer, renal carcinoma, NSCLC, melanoma.

HRD is an index for measuring functional defects in homologous recombination DNA repair, deriving from germline or somatic mutations in BRCA1/2 function or other mechanisms [47]. Preclinical data showed that *SETD2* is required for HRR, however we observed unimpaired HRR in certain types of cancers in *SETD2* deleterious mutant samples. For synthetical lethal was found in cells with deficient of both BER and HRR deficient patients we postulate that tumors cells with deficient in both MMR and HRR will undergo cell death but further scientific evidence is needed to confirm this conjecture [48]. The remaining tumor cells that harbor mutations in one repair pathway would, consequently, have a lower HRD score. Meanwhile, the dysfunction of HRR is considered to be a biomarker for PARPi and platinum-based chemotherapy. Therefore, these results indicate that ICI treatment rather than chemotherapy or target therapy may be more suitable for endometrial carcinoma, colorectal adenocarcinoma, and stomach adenocarcinoma patients with *SETD2* mutations.

Extrinsic and intrinsic immune escape is crucial in tumorigenesis and cancer progression. Four reasons account for extrinsic immune escape: lack of immune cells, presence of immunoinhibitory cells, such as type 2 macrophages and regulatory T-cells (T_reg_); high concentrations of immunoinhibitory cytokines, such as interleukin 10 (IL10) and transforming growth factor β (TGF-β); and fibrosis [49]. Also, it is known that at least two aspects, including tumor immunogenicity and expression of immune checkpoint molecules, are responsible for intrinsic immune escape [50]. When analyzing pooled data from patients with different cancer types, our results showed higher levels of infiltrating of CD8 cells, lower levels of infiltrating of M2 macrophages, higher TMB, and higher expression of immune checkpoint molecules in the *SETD2* deleterious mutation group. This may be the underlying mechanism for a favorable ICI response in this group. When comparing the differences across cancer types, our results showed that a more inflamed tumor microenvironment was present in the *SETD2* deleterious mutation group in colorectal carcinoma, endometrial carcinoma, and renal cell carcinoma. The data above illustrate the underlying mechanisms for the favored ICI response in the *SETD2* deleterious mutation group.

By analyzing our 13 patients with ICI treatment, we found a better ORR in these patients than in non-selective cancer patients treated with immunotherapy. Furthermore, we verified that *SETD2* deleterious mutations may have the potential to serve as a biomarker for ICI therapy. Although the survival advantage has been shown in the MSKCC-IO cohort, there are limitations to using local cohorts for survival analysis like the insufficient sample size, lack of control group and the combined treatment which can’t rule out the possibility that the survival benefit of *SETD2* mutation may result from chemotherapy or tyrosine kinase inhibitors; as such, a large randomized prospective study will be needed to further test our hypothesis. Additionally, the specific pathway through which *SETD2* affects prognosis is not made clear in our study; therefore, in vivo and in vitro experiments are needed.

This study is of great importance. First, with respect to response, MSH-H/dMMR performed far better than other biomarkers, with ORR reaching 53% compared with just 29% in TMB-H patients. Based on our finding that *SETD2* influences MSI-H with the adjustment of MMR related proteins, we recommend that *SETD2* should be considered when testing MMR function by IHC (in addition to the classical MSH2, MSH6, MLH1, and PMS2 screening). This will screen for more groups that may benefit from ICI treatment. Second, TMB has been approved by the FDA as a tissue agnostic biomarker for ICI treatment. We found that *SETD2* influences survival outcome independently of TMB following ICI treatment in pancancer patients, and also has a much lower death hazard compared to TMB. Third, although preclinical data has shown that *SETD2* deficient cells have impaired MMR and HRR, we identified unimpaired HRR in endometrial carcinoma, colorectal adenocarcinoma, and stomach adenocarcinoma; this may be explained by possible synthetic lethality between impaired HRR and impaired MMR. Fourth, a *SETD2* mutation rate greater than 5% was found in 10 cancer types, indicating that a large population of patients may benefit from ICI treatment. Taken together, our data demonstrate that *SETD2* should be given more attention as a candidate biomarker for ICI treatment.

## Conclusion

Based on our finding that *SETD2* dysfunction affects ICI treatment prognosis independently of TMB-H and has a lower death hazard than TMB-H in pancancer patients, *SETD2* has the potential to serve as a biomarker for ICI treatment. Additionally, in endometrial carcinoma, colorectal adenocarcinoma, and stomach adenocarcinoma, *SETD2* should be considered when dMMR is detected by immunohistochemistry.

## Supplementary Information

**Additional file 1.**

## Data Availability

The data supporting the conclusions of this article are included within the article and the additional files. The original case report forms and other related datasets are available from the corresponding author on reasonable request.

## References

[1] Tang J, Shalabi A, Hubbard-Lucey VM (2018). Comprehensive analysis of the clinical immuno-oncology landscape. Ann Oncol.

[2] Couzin-Frankel J (2013). Breakthrough of the year 2013. Cancer immunotherapy. Science.

[3] Ribas A, Wolchok JD (2018). Cancer immunotherapy using checkpoint blockade. Science.

[4] Le DT, Durham JN, Smith KN (2017). Mismatch repair deficiency predicts response of solid tumors to PD-1 blockade. Science.

[5] Weber JS, Hodi FS, Wolchok JD (2017). Safety profile of nivolumab monotherapy: a pooled analysis of patients with advanced melanoma. J Clin Oncol.

[6] Bertrand A, Kostine M, Barnetche T, Truchetet M-E, Schaeverbeke T (2015). Immune related adverse events associated with anti-CTLA-4 antibodies: systematic review and meta-analysis. BMC Med.

[7] Wolchok JD, Chiarion-Sileni V, Gonzalez R (2017). Overall survival with combined nivolumab and ipilimumab in advanced melanoma. N Engl J Med.

[8] Gibney GT, Weiner LM, Atkins MB (2016). Predictive biomarkers for checkpoint inhibitor-based immunotherapy. Lancet Oncol.

[9] Bonneville R, Krook MA, Kautto EA (2017). Landscape of microsatellite instability across 39 cancer types. JCO Precis Oncol.

[10] Marabelle A, Fakih M, Lopez J (2020). Association of tumour mutational burden with outcomes in patients with advanced solid tumours treated with pembrolizumab: prospective biomarker analysis of the multicohort, open-label, phase 2 KEYNOTE-158 study. Lancet Oncol.

[11] Mouw KW, Goldberg MS, Konstantinopoulos PA, D'Andrea AD (2017). DNA damage and repair biomarkers of immunotherapy response. Cancer Discov.

[12] Chen Y, Chen G, Li J (2019). Association of tumor protein p53 and ataxia-telangiectasia mutated comutation with response to immune checkpoint inhibitors and mortality in patients with non-small cell lung cancer. JAMA Netw Open.

[13] Campbell BB, Light N, Fabrizio D (2017). Comprehensive analysis of hypermutation in human cancer. Cell.

[14] Mehnert JM, Panda A, Zhong H (2016). Immune activation and response to pembrolizumab in POLE-mutant endometrial cancer. J Clin Invest.

[15] Huang Y, Gu L, Li GM (2018). H3K36me3-mediated mismatch repair preferentially protects actively transcribed genes from mutation. J Biol Chem.

[16] Li F, Mao G, Tong D (2013). The histone mark H3K36me3 regulates human DNA mismatch repair through its interaction with MutSalpha. Cell.

[17] Pfister SX, Ahrabi S, Zalmas LP (2014). SETD2-dependent histone H3K36 trimethylation is required for homologous recombination repair and genome stability. Cell Rep.

[18] Carvalho S, Vitor AC, Sridhara SC (2014). SETD2 is required for DNA double-strand break repair and activation of the p53-mediated checkpoint. Elife.

[19] Dalgliesh GL, Furge K, Greenman C (2010). Systematic sequencing of renal carcinoma reveals inactivation of histone modifying genes. Nature.

[20] Chen R, Zhao W-Q, Fang C, Yang X, Ji M (2020). Histone methyltransferase SETD2: a potential tumor suppressor in solid cancers. J Cancer.

[21] Hutter C, Zenklusen JC (2018). The cancer genome atlas: creating lasting value beyond its data. Cell.

[22] Samstein RM, Lee CH, Shoushtari AN (2019). Tumor mutational load predicts survival after immunotherapy across multiple cancer types. Nat Genet.

[23] Zehir A, Benayed R, Shah RH (2017). Mutational landscape of metastatic cancer revealed from prospective clinical sequencing of 10,000 patients. Nat Med.

[24] Zehir A, Benayed R, Shah RH (2017). Mutational landscape of metastatic cancer revealed from prospective clinical sequencing of 10,000 patients. Nat Med.

[25] Niu B, Ye K, Zhang Q (2014). MSIsensor: microsatellite instability detection using paired tumor-normal sequence data. Bioinformatics.

[26] Mandal R, Samstein RM, Lee K-W (2019). Genetic diversity of tumors with mismatch repair deficiency influences anti-PD-1 immunotherapy response. Science.

[27] Bailey MH, Tokheim C, Porta-Pardo E (2018). Comprehensive Characterization of Cancer Driver Genes and Mutations. Cell.

[28] Ding L, Bailey MH, Porta-Pardo E (2018). Perspective on oncogenic processes at the end of the beginning of cancer genomics. Cell.

[29] Thorsson V, Gibbs DL, Brown SD (2018). The Immune Landscape of Cancer. Immunity..

[30] Newman AM, Liu CL, Green MR (2015). Robust enumeration of cell subsets from tissue expression profiles. Nat Methods.

[31] Yoshihara K, Shahmoradgoli M, Martinez E (2013). Inferring tumour purity and stromal and immune cell admixture from expression data. Nat Commun.

[32] Li B, Dewey CN (2011). RSEM: accurate transcript quantification from RNA-Seq data with or without a reference genome. BMC Bioinformatics.

[33] Barbie DA, Tamayo P, Boehm JS (2009). Systematic RNA interference reveals that oncogenic KRAS-driven cancers require TBK1. Nature.

[34] Core Team R (2013). R: a language and environment for statistical computing.

[35] Kim T-M, Laird PW, Park PJ (2013). The landscape of microsatellite instability in colorectal and endometrial cancer genomes. Cell.

[36] Marabelle A, Fakih M, Lopez J (2020). Association of tumour mutational burden with outcomes in patients with advanced solid tumours treated with pembrolizumab: prospective biomarker analysis of the multicohort, open-label, phase 2 KEYNOTE-158 study. Lancet Oncol.

[37] Herbst RS, Baas P, Kim D-W (2016). Pembrolizumab versus docetaxel for previously treated, PD-L1-positive, advanced non-small-cell lung cancer (KEYNOTE-010): a randomised controlled trial. Lancet.

[38] Muro K, Chung HC, Shankaran V (2016). Pembrolizumab for patients with PD-L1-positive advanced gastric cancer (KEYNOTE-012): a multicentre, open-label, phase 1b trial. Lancet Oncol.

[39] Weber JS, D'Angelo SP, Minor D (2015). Nivolumab versus chemotherapy in patients with advanced melanoma who progressed after anti-CTLA-4 treatment (CheckMate 037): a randomised, controlled, open-label, phase 3 trial. Lancet Oncol.

[40] Ferris RL, Blumenschein G, Fayette J (2016). Nivolumab for recurrent squamous-cell carcinoma of the head and neck. N Engl J Med.

[41] Larkin J, Hodi FS, Wolchok JD (2015). Combined nivolumab and ipilimumab or monotherapy in untreated melanoma. N Engl J Med.

[42] Motzer RJ, Escudier B, McDermott DF (2015). Nivolumab versus everolimus in advanced renal-cell carcinoma. N Engl J Med.

[43] Tomasova K, Cumova A, Seborova K (2020). DNA repair and ovarian carcinogenesis: impact on risk, prognosis and therapy outcome. Cancers (Basel).

[44] Cleary JM, Aguirre AJ, Shapiro GI, D'Andrea AD (2020). Biomarker-Guided Development of DNA Repair Inhibitors. Mol Cell.

[45] Park VS, Pursell ZF (2019). POLE proofreading defects: Contributions to mutagenesis and cancer. DNA Repair.

[46] Nicolas E, Golemis EA, Arora S (2016). POLD1: Central mediator of DNA replication and repair, and implication in cancer and other pathologies. Gene.

[47] Turner N, Tutt A, Ashworth A (2004). Hallmarks of 'BRCAness' in sporadic cancers. Nat Rev Cancer.

[48] Basu B, Sandhu SK, de Bono JS (2012). PARP inhibitors: mechanism of action and their potential role in the prevention and treatment of cancer. Drugs.

[49] Spranger S (2016). Mechanisms of tumor escape in the context of the T-cell-inflamed and the non-T-cell-inflamed tumor microenvironment. Int Immunol.

[50] Schreiber RD, Old LJ, Smyth MJ (2011). Cancer immunoediting: integrating immunity's roles in cancer suppression and promotion. Science.

