# Supplementary material for: SETD2 variation correlates with tumor mutational burden and MSI along with improved response to immunotherapy
Source: BMC Cancer. 2023 Jul 21;23:686. doi: 10.1186/s12885-023-10920-4 (PMC10360270; doi:10.1186/s12885-023-10920-4)
Supplement: Supplementary file 1 — Additional file 1. [file 12885_2023_10920_MOESM1_ESM.docx]

Supplementary information of article entitled “SETD2 variation correlates with tumor mutational burden and MSI along with improved response to immunotherapy.”

Figure S1: Comparison of the ssGSEA between the *SETD2* non-deleterious mutation group and the deleterious mutation group in patients with seven types of cancer in the TCGA cohort including bladder urinary cancer, colorectal adenocarcinoma, renal carcinoma, non-small cell lung cancer, skin cutaneous melanoma, stomach adenocarcinoma, and endometrial carcinoma. (Wilcoxon rank-sum test, ****P<0.0001; ***P <0.001; **P<0.01; *P<0.05 Abbreviations: NSCLC, Non-small cell lung cancer; HRR, homologous recombination repair; MMR, mismatch repair; BER, base excision repair; NER, nucleotide excision repair; FA, Fanconi anemia; TLS, translesion DNA synthesis; NHEJ, non-homologous end-joining; CPF, checkpoint factors.)


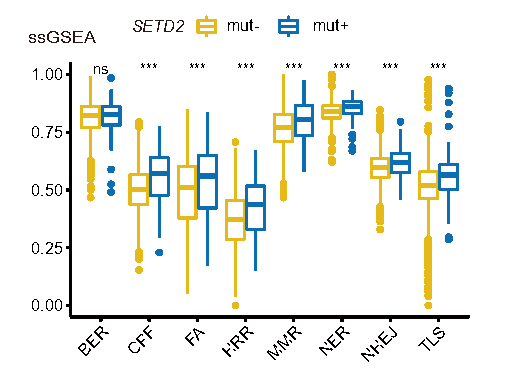


Figure S2: Correlation between *SETD2* deleterious mutations and MSI (A-B) The difference of MSIsensor score in different molecular subtypes in endometrial carcinoma, colorectal carcinoma and stomach adenocarcinoma . (Wilcoxon rank-sum test). (C) The coincident and exclusive associations across mutated genes (Fisher’s exact test,▪ p<0.05; * p<0.001). Abbreviations: OR, odd ratio; mut+, deleterious mutation; mut-, non-deleterious mutation; *MMRS, MSH2, MSH6, PMS2, or MLH1; MMRS*or*SETD2, MSH2, MSH6, PMS2, MLH1* or *SETD2)*


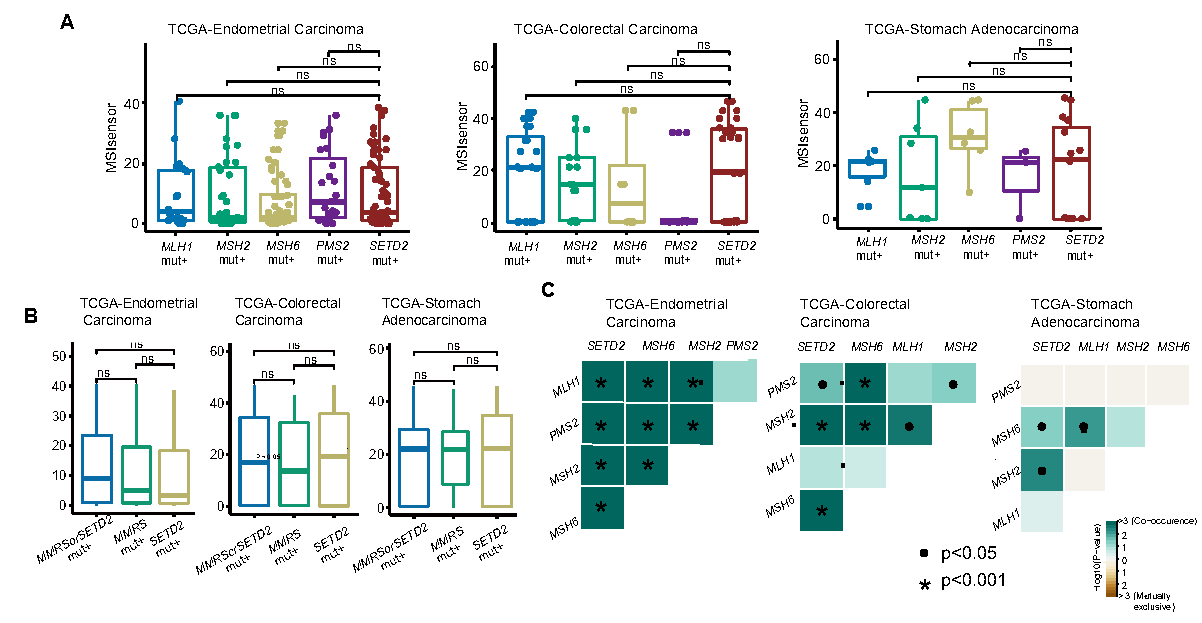


**Figure S3.** Difference of ESTIMATE algorithm-derived immune scores between the SETD2 deleterious mutation and SETD2 non-deleterious groups in different cancer types in the TCGA cohort.


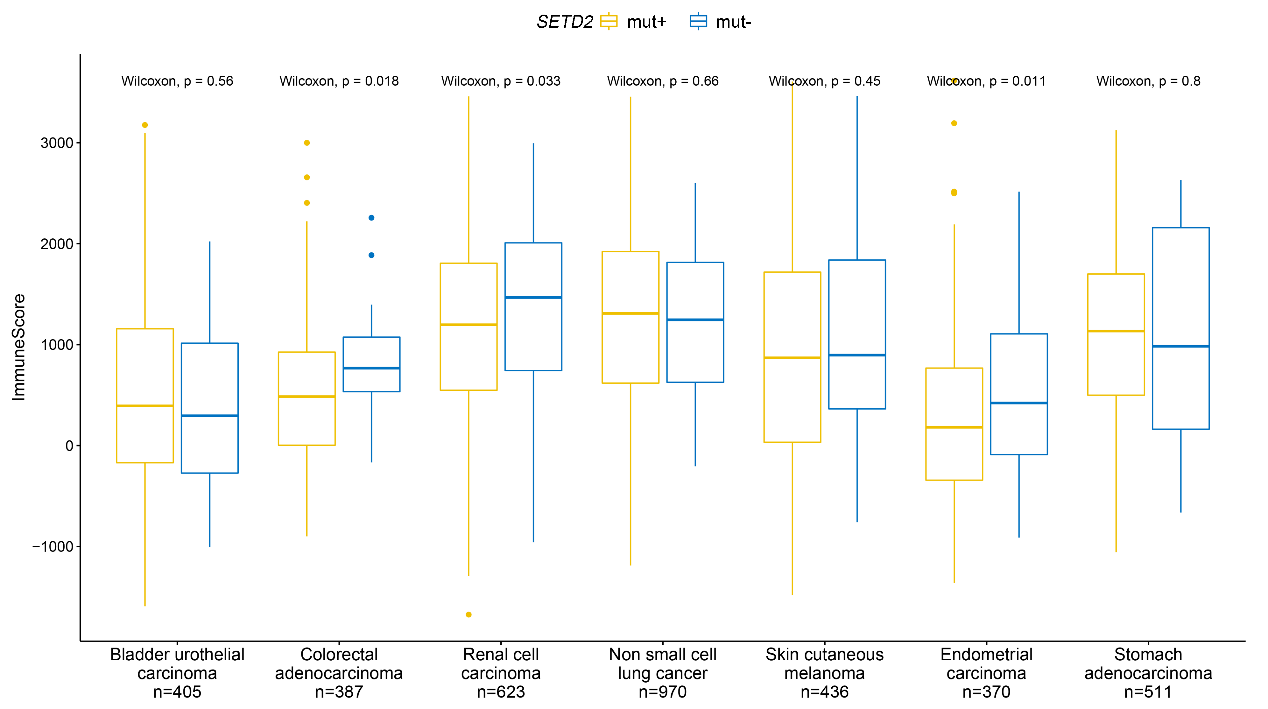


Figure S4: Comparison of the expression of immune-related gene profiles between *SETD2* non-deleterious mutation group and deleterious mutation group in patients of renal carcinoma (A), colorectal adenocarcinoma (B), and endometrial carcinoma.


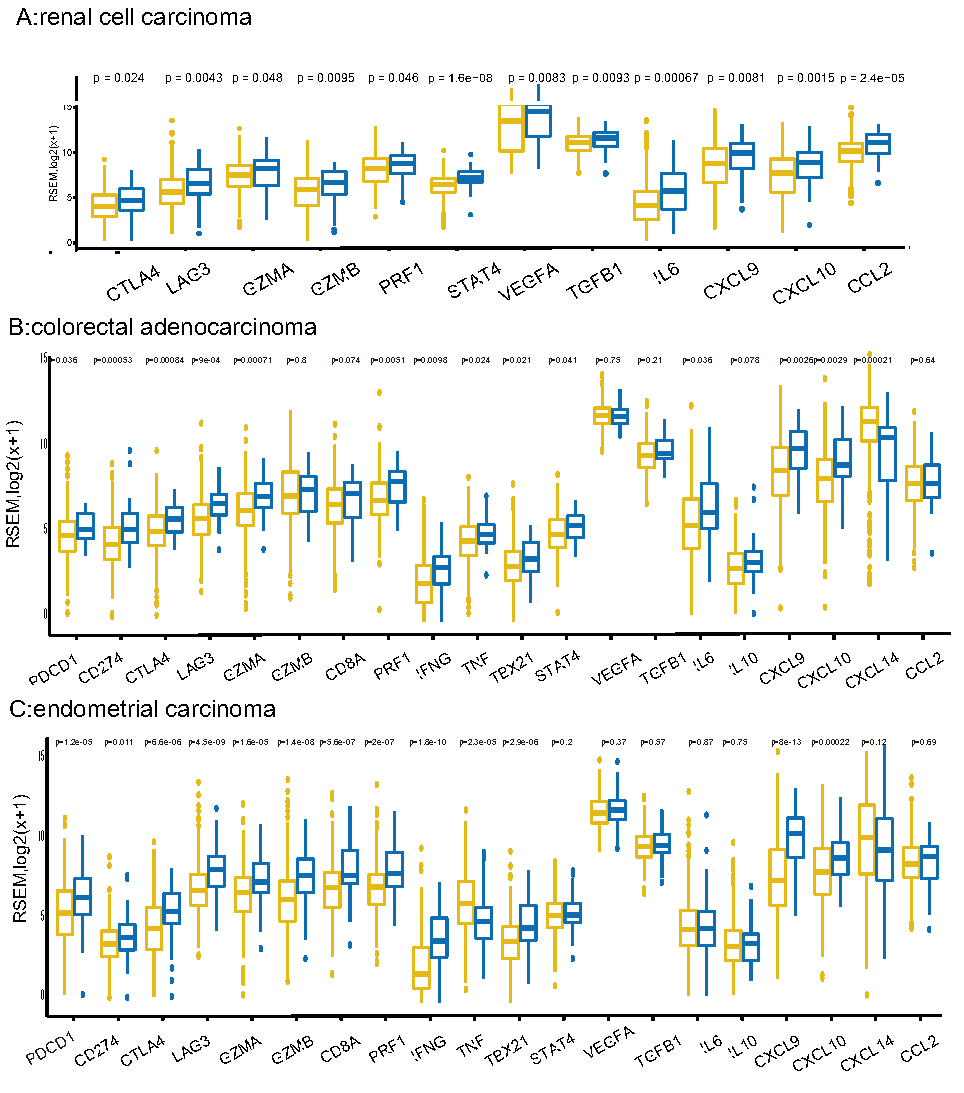


Figure S5: Disease course and clinical response in two patients with exceptional response to Nivolumab-based immunotherapy.

**
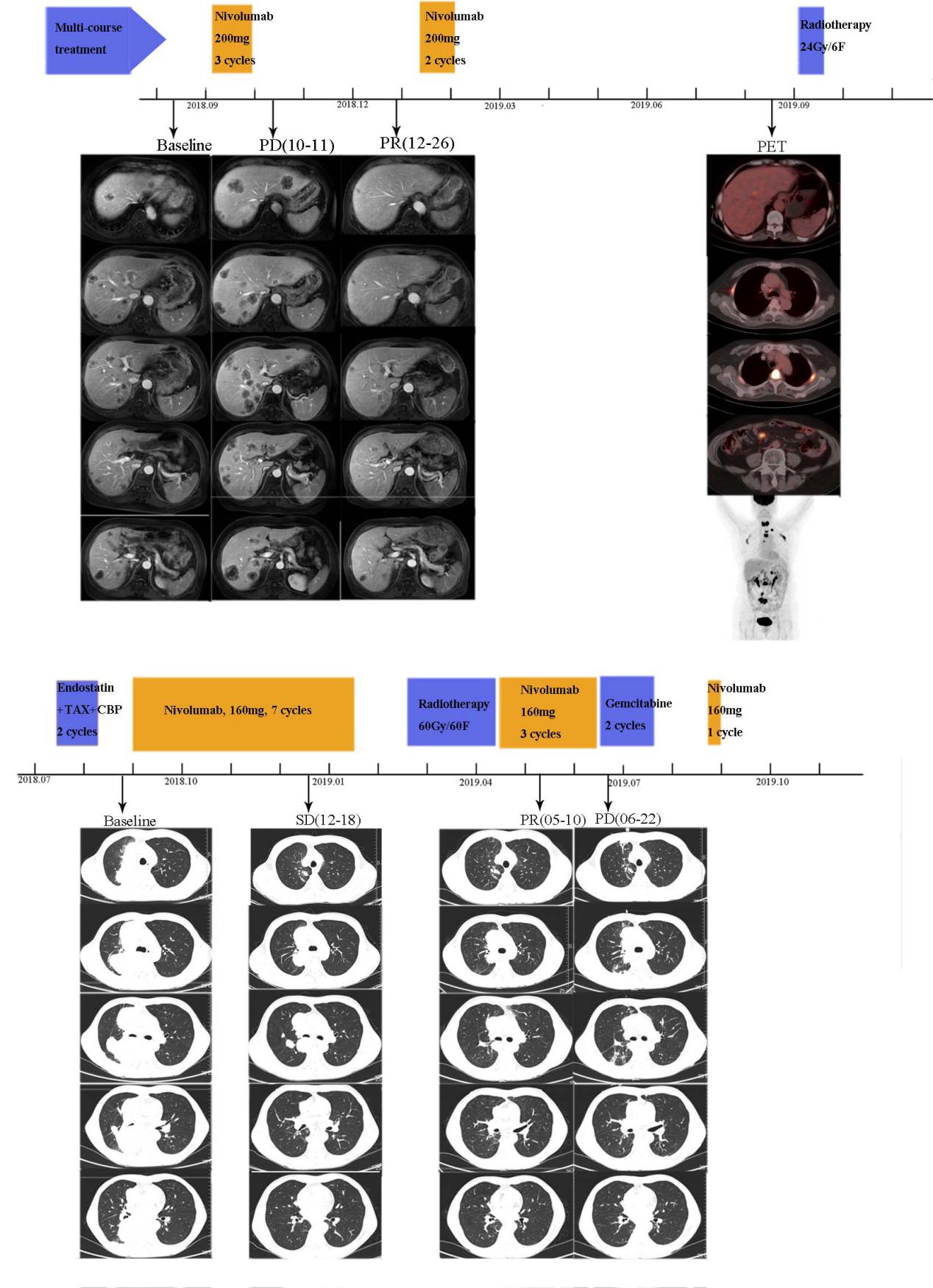
**

Table S1. The cancer types and number of samples included in Geneplus pan-cancer cohort.

| **Cancer types** | **The number of samples** |
| --- | --- |
| Melanoma | 136 |
| Small cell lung cancer | 88 |
| Cervical cancer | 116 |
| Urothelial carcinoma | 75 |
| Colorectal adenocarcinoma | 1156 |
| Prostate Cancer | 52 |
| Breast cancer | 261 |
| Non-small cell lung cancer | 3630 |
| Gastric Cancer | 287 |
| Hepatocellular Carcinoma | 195 |
| Glioblastoma | 215 |
| Ovarian Cancer | 233 |
| Pancreatic Cancer | 189 |
| Renal Cell Carcinoma | 93 |
| Total | 6726 |

Table S2： Detailed list of genes in the geneplus 1021 Panel

| **Gene list of 1021 panel** |
| --- |
| ABL1 ABL2 AKT1 AKT2 AKT3 ALK APC AR ARAF ATM ATR AURKA AURKB AXL BAP1 BCL2 BRAF BRCA1 BRCA2 BRD2 BRD3 BRD BTK C11orf30 C1QA C1S CBL CCND1 CCND2 CCND3 CCNE1 CD27 CDH1 CDK13 CDK CDK CDK8 CDKN1A CDKN1B CDKN2A CDKN2B CHEK1 CHEK2 CRKL CSF1R CTNNB1 DDR1 DDR2 DNMT3A EGFR EPHA2 EPHA3 EPHA5 ERBB2 ERBB3 ERBB ERCC1 ERG ESR1 EZH2 FAT1 FBXW7 FCGR2A FCGR2B FCGR3A FGFR1 FGFR2 FGFR3 FGFR FLCN FLT1 FLT3 FLT FOXA1 FOXL2 GAB2 GATA3 GNA11 GNAQ GNAS HDAC1 HDAC HGF HRAS IDH1 IDH2 IGF1R IL7R INPP B IRS2 JAK1 JAK2 JAK3 KDR KIT KRAS MAP2K1 MAP2K2 MAPK1 MAPK3 MCL1 MDM2 MDM MED12 MET MITF MLH1 MLH3 MPL MS A1 MSH2 MSH3 MSH MTOR MYC MYD88 NF1 NF2 NOTCH1 NOTCH2 NOTCH3 NOTCH NRAS NTRK1 NTRK3 PALB2 PDGFRA PDGFRB PDK1 PIK3CA PIK3CB PIK3R1 PIK3R2 PMS1 PMS2 PRKAA1 PSMB1 PSMB5 PTCH1 PTCH2 PTEN PTPN11 RAF1 RARA RB1 RET RHEB RHOA RICTOR RNF 3 ROCK1 ROS1 RPS KB1 SMARCA SMARCB1 SMO SRC STAT1 STAT3 STK11 SYK TMPRSS2 TOP1 TP53 TSC1 TSC2 EGFA HL XPO1 XRCC1 KEAP1 NFE2L2 REG1B TPTE CSMD3 FAM135B U2AF1 THSD7A MLL3 EYA HCN1 AKR1B10 SLC A5 DPP10 SCN7A SNTG1 PS13A IL1RAPL1 CTNNA2 FAM5C CACNA1E KRTAP5-5 PDE1C RYR2 NRXN1 COL19A1 LRP1B GKN2 CD5L SPTA1 DHX9 ADAMTS20 NLRP CDH18 MYH2 OR5L2 OR A15 OR F1 OR C OR2T PSG2 ITM2A TNN OCA2 CNTN5 POM121L12 LRRC7 CNTNAP5 SLC A10 GFRAL SORCS3 POTEG F9 SLC2 A3 UNC5D PDE DIP MRPL1 COL25A1 TNR GALNT13 EIF3E SLC5A1 COASY TBX15 PYHIN1 PSG5 BTRC MDGA2 GUCY1A3 TIMD AK5 ODZ3 COL5A2 NTM LTBP1 PRSS1 CNGB3 SI TMEM132D ASTN1 SAGE1 ADAMTS12 EPB 1L B POLR3B ATP10B CSMD1 FBN2 EXOC5 ANKRD30A TRIML1 POLDIP2 KLHL1 TRIM58 GRIA3 CNOT NA 3 TRPC5 LRRC2 ADAMTS1 ACER2 AMOT OBP2A INHBA PTPRD FAM21A RUNX1 FAM157B SLC8A1 CBFB C9orf 3 TBP NBPF10 NRXN2 TAF1B PTCD3 ACTL B SH3PXD2A PRKAG3 UCK2 DLST MEF FDCSP COPA LMBR1L CD99 RBMX ZDHHC11 MGAM COL1 A1 PPA1 APLP2 ELL3 LILRB3 KIR2DL3 ENTPD BAX ANKRD3 B LRP2 SF3B1 CEACAM20 C19orf38 TBX3 UMOD LPHN3 FRG1 IFT172 ZBTB8OS TNNT1 TNFAIP USP12 SGIP1 TEX35 WASL NWD1 MAP K1 MAPRE3 HAAO SEC1 L CD9 PIWIL1 CLEC1 A DPP GMDS ST18 DKC1 FOLH1 SPAG1 CACNA1D CSPP1 PAGE1 BRWD3 CDH2 ABCA8 TMC2 EFHA2 HAUS WLS TNFSF DDB1 CTSF TMX3 MICALL1 NUDCD2 PRKDC PREX2 BCAS2 THOC1 LILRB PGAP1 CPA1 FAM3A RPL22 NBPF1 RYR3 PTPLAD1 FNDC METTL5 CAMKK1 NCOR1 COL1A1 EZF1 KLHL1 PDRG1 ILL COL A DMXL1 ADAMTS19 SYCP2L EPB 1L2 TNS3 IKBKAP COL5A1 FATE1 MORN1 MAEL SLC38A ATP 0A2 CASC TIMP3 DOCK3 KDM A CASQ2 CDK18 FRMD A TRUB1 SLCO1B7 |

Table S3. Gene list of immune-related gene set.

| **Gene set** | **Gene** |
| --- | --- |
| Checkpoint | *PDCD1, CD274, CTLA4, LAG3* |
| CTL (cytotoxic lymphocyte) | *GZMA, GZMB, CD8A, PRF1* |
| Th1 | *IFNG, TNF, TBX21, STAT4* |
| Anti-inflammatory cytokines | *VEGFA, TGFB1,IL6 , IL10* |
| Anti-tumor-chemokines | *CXCL9, CXCL10, CXCL14* |
| Pro-tumor-chemokines | *CCL3* |
|  |  |

Table S4. Clinical characteristics of 13 patients.

Table S5: Univariable and Multivariable Analyses of Overall Survival in the MSKCC cohort.

| **Parameters** | | **N** |  | **Univariable Analysis** | | |  | **Multivariable Analysis** | | |
| --- | --- | --- | --- | --- | --- | --- | --- | --- | --- | --- |
|  |  |  |  | **HR** | **95%CI** | **P** |  | **HR** | **95%CI** | **P** |
| **Age** | <=60 | 749 |  | 1.002 | 0.874-1.148 | .979 |  |  | | |
|  | >60 | 912 |  |  |  |  |  |  |  |  |
| **Sex** | Female | 627 |  | 0.882 | 0.767-1.014 | .078 |  |  |  |  |
|  | Male | 1034 |  |  |  |  |  |  |  |  |
| **TMB** | TMB-low | 1235 |  | 0.694 | 0.587-0.821 | .000 |  | 0.757 | 0.634-0.905 | .002 |
|  | TMB-high | 426 |  |  |  |  |  |  |  |  |
| **DRUG_TYPE** | PD-1/PDL-1 | 1307 |  |  |  |  |  |  |  |  |
|  | CTLA4 | 99 |  | 0.577 | 0.434-0.768 | .000 |  | 0.566 | 0.425-0.753 | .000 |
|  | Combo | 255 |  | 0.531 | 0.428-0.658 | .000 |  | 0.519 | 0.418-0.644 | .000 |
| ***SETD2*** | mut- | 1552 |  | 0.455 | 0.322-0.641 | .000 |  | 0.511 | 0.360-0.725 | .000 |
|  | mut+ | 109 |  |  |  |  |  |  |  |  |
| ***MMR* genes** | mut- | 1576 |  | 0.645 | 0.448-0.929 | .019 |  | 0.873 | 0.595-1.281 | .488 |
|  | mut+ | 85 |  |  |  |  |  |  |  |  |
| ***POLE/D1*** | mut- | 1576 |  | 0.675 | 0.471-0.966 | .032 |  | 0.804 | 0.553-1.169 | .253 |
|  | mut+ | 85 |  |  |  |  |  |  |  |  |
| ***BRCA1/2*** | Mut- | 1554 |  | 0.818 | 0.611-1.095 | .177 |  |  | | |
|  | Mut+ | 107 |  |  |  |  |  |  |  |  |

***GenePlus-Beijing Clinical Sequencing cohort***

Data of Chinese pan-cancer population were involved from GenePlus-Beijing Clinical Sequencing cohort from March,2018 to April,2020 and was introduced as follows.

1. **Specimen processing and DNA extraction**

The genomic DNA from frozen tissue samples was extracted by using the Tissue gDNA exaction Kit（Qiagen, Hilden, Germany), FFPE(formalin-fixed, paraffin-embedded) DNA was isolated by using a commercially available kit (Maxwell® 16 FFPE Plus LEV DNA Purification, Qiagen, Hilden, Germany Kit. catalog: AS1135). The DNA concentration was measured using a Qubit fluorometer and the Qubit dsDNA HS (High Sensitivity) Assay Kit (Invitrogen, Carlsbad, CA, USA).

1. **Library preparation, target capture and next-generation sequencing.**

Sequencing was carried out using 2 × 75-bp paired-end reads on an Illumina instrument according to the manufacturer’s recommendations using the KAPA DNA Library Preparation Kit (Kapa Biosystems, Wilmington, MA, USA) or 2 × 100-bp paired-end reads on Gene+seq sequencer instrument and libraries constructed using NEBNext® Ultra™ II DNA Library Prep Kit (New England Biolabs, Ipswich, MA, USA).

Barcoded libraries were hybridized to a customized panel of 1021 genes containing whole exons and selected introns of 288 common driver genes and high-frequently mutant regions recorded in the Catalogue of Somatic Mutations in Cancer (COSMIC, http://cancer.sanger.ac.uk/cosmic) were added of 733 genes.

1. **Somatic mutation calling**

Somatic single-nucleotide variations (SNVs) were called using the MuTect2 algorithm (https://software.broadinstitute.org/gatk/documentation/tooldocs/3.8-0/org_broadinstitute_gatk_tools_walkers_cancer_m2_MuTect2.php). Insertions or deletions of small fragments (Indels) were called using MuTect2 with default parameters. Variants detected in matched control samples with three or more reads indicating Indels at the same location or in the 40-bp flanking regions of experimental samples or residing near regions with low complexity or short tandem repeats were removed.

Copy number variants (CNVs) of ERBB2 were detected using the Contra algorithm (<http://contra-cnv.sourceforge.net>).
